# The effect of β-adrenergic blockade and COX-2 inhibition on healing of colon, muscle, and skin in rats undergoing colonic anastomosis 

**DOI:** 10.5414/CP201550

**Published:** 2011-08-11

**Authors:** O. Hazut, L. Shaashua, M. Benish, B. Levi, L. Sorski, B. Benjamin, A. Hoffman, O. Zmora, S. Ben-Eliyahu

**Affiliations:** * Both contributed equally; 1 NeuroImmunology Research Unit, Department of Psychology, Tel Aviv University,; 2 Department of Surgery, Meir Medical Center, Tel Aviv, and; 3 Department of Surgery and Transplantation, Sheba Medical Center, Tel-Hashomer, Israel

**Keywords:** COX-2 inhibitors, β-adrenergic blockers, colonic anastomosis, perioperative

## Abstract

**Abstract.** Objective: COX inhibitors and b-adrenergic blockers were recently shown to reduce cancer progression in animal models through various mechanisms. These include the prevention of immune suppression during the critical perioperative period, and the preclusion of direct promoting effects of catecholamines and prostaglandins on malignant tissue growth. To assess the safety of such pharmacological treatments in the context of oncologic surgery, the current study evaluates wound healing efficacy in the skin, muscle, and colon tissues in rats undergoing colonic anastomosis. Methods: F344 rats were treated daily with a COX-2 inhibitor (etodolac), a b-adrenergic blocker (propranolol), both drugs or vehicles. All rats underwent skin punch biopsy, and half were also subjected to laparotomy and colonic anastomosis. Tensile strength of the abdominal wall and colonic bursting pressure were assessed on Days 3, 7, and 30 postoperatively, and skin biopsy site healing was scored on Days 2, 4, and 6 postoperatively. Results: None of the drug treatments produced any deleterious effects along the expected course of tissue healing. On Day 30, colon bursting pressure showed an abnormal strengthening in animals undergoing anastomosis compared to non-operated animals, across all drug treatments. This abnormal strengthening was attenuated by etodolac. In the skin, surgery reduced healing rate, irrespective of drug treatments. Conclusions: Effective doses of etodolac and propranolol caused no negative effects on wound healing processes in rats. The apparent safety of such treatments, together with their potential clinical benefits, suggests the incorporation of these treatments in oncologic patients undergoing curative tumor resection.

## Introduction 

Surgery to excise the primary tumor is crucial in the great majority of cancer patients presenting solid tumors. While this treatment is beneficial in removing the major mass of malignant cells, the surgical stress, and other aspects of tumor removal, have been suggested to promote cancer recurrence [1, 2, 3, 4, 5, 6, 7, 8]. Recently we have presented evidence in animal models that a perioperative drug treatment, based on a combined administration of a cyclo-oxygenase (COX)-2 inhibitor and a b-adrenergic blocker (but neither drug alone), has marked beneficial effects: it reduces postoperative immunosuppression, increases postoperative resistance to metastases, and improves postoperative long term survival rates in several models of spontaneous metastasis [9, 10, 11, 65]. However, critical to any surgical procedure is an effective process of tissue healing, which may be affected by perioperative administration of drugs. Thus, the drug treatment presented above should be tested in light of this criterion before being used in cancer patients.

The wound healing process is comprised of three major phases: inflammation, proliferation, and remodeling [12]. Efficient and quick wound healing is crucial in operated tissues, specifically in the gastrointestinal tract. Failure of intestinal anastomosis, which usually occurs during the first postoperative week [13, 14, 15], is associated with increased morbidity and mortality [16, 17]. 

COX inhibitors and b-blockers are commonly administered in the perioperative period, mainly for purposes of pain alleviation [18, 19] and attenuation of excess stress-associated symptoms [20, 21], respectively. Of the three COX isoforms, COX-2 is the prominent source of prostaglandins (PGs) in response to surgery and stress, mediating pain and inflammation [22]. It was suggested that the inflammatory phase of the wound healing is paced by several factors, including PGs and catecholamines (CAs) [23, 25]. Therefore, COX-2 inhibitors and b-blockers may potentially affect wound healing. 

Studies regarding the effects of COX-2 inhibition on wound healing revealed inconsistent results. Some studies reported no negative effects of COX-2 inhibition on healing of various tissues [26, 27, 28]. For example, Gilroy et al. [29] found that COX-2 did not cause mucosal damage in the intestine. In the context of surgery, COX-2 was reported to produce less negative effects than the widely used non-selective NSAIDs [30, 31, 32]. On the other hand, COX-2 is involved in bone [33, 34] and skin [35] wound healing, and several reports suggested that COX-2 blockade is harmful in this context [36]. Similarly, evidence indicated that COX-2 is involved in intestinal wound healing [37, 38, 39], and its blockade was reported to interfere with this process [41, 41]. The literature regarding the impact of b-blockade on wound healing is scant, and it was reported to improve skin wound healing by reducing local inflammatory response [42, 43]. 

In the current study in rats we tested the effects of the COX-2 inhibitor, etodolac, and the b-blocker, propranolol, separately and in combined administration, on colonic bursting pressure and abdominal muscle tissue strength at different time intervals following laparotomy and colonic anastomosis. Skin healing following standard biopsy was simultaneously assessed. The safety of these specific drugs is tested to allow their use in colon cancer patients undergoing tumor resection with curative intent. 

## Methods 

### Surgical procedures

All rats were subjected to skin punch biopsy, and 170 rats, equally distributed in the drug treatment blocks, concomitantly underwent laparotomy and colonic anastomosis. 

**Punch biopsy**

Animals were anesthetized with 2.5% isoflurane, a 2 × 2 cm area of their flank skin was shaved (2 cm right to the vertebrate column in mid distance from the front and the back limbs). A skin punch was made throughout all three layers of the skin by a standard 3 mm diameter punch needle (Miltex, Inc., Painsboro, NJ, USA). The wound was examined for abnormal bleeding and shape. 

**Laparotomy and colonic anastomosis**

Under general anesthesia using 2.5 – 3.5% isoflurane, a 4 cm midline abdominal incision was made, and the descending colon was exposed. A transverse incision of 180° was made in the descending colon, 3 cm above the peritoneal reflection. The incision was then sutured (hand-sewn) using interrupted 5/0 monofilament polypropylene sutures. Colonic anastomoses were performed by trained surgeons, with the aid of magnification glass. During the procedure, phosphate buffered saline (PBS) was repeatedly instilled on the exposed colon. At the completion of the procedure, the abdominal wall (including the muscle and skin) was closed by mass closure using continuous 3/0 nylon sutures. 

### Assessment of tissue strength

All animals were randomly assigned within the treatment blocks for sacrifice by an overdose of isoflurane at 3, 7 or 30 days following the surgical procedure (skin biopsy with or without laparotomy and anastomosis). Assessment of the healing process in the different tissues was carried out by investigators blind to the group allocation. 

### Abdominal wall strength assessment – tensile strength

Abdominal wall strength was assessed only in animals undergoing laparotomy and colonic anastomosis. A square of 2 × 2 cm of the abdominal wall muscle and skin, 1 cm aside of the incision line bilaterally, was excised. The nylon suture thread was carefully removed, and each end of the excised section was placed in a tensiometer (parallel to the suture, at a distance of 1 cm from it). Strength on the incision was gradually increased at a constant rate, and tensile strength was defined as the weight in which the banks of the scar were completely separated. 

**Colon strength assessment – bursting pressure**

At the time of sacrifice, a laparotomy was performed and the abdominal cavity was explored for evidence of anastomotic leak or bowel obstruction. A colonic section of approximately 4 cm, with the anastomosis at its midportion, was excised. A silicon catheter (4 mm in diameter) was tightly secured to one end of the lumen, and the other end was sealed by a hemostat. An electronic continuous syringe pump (Carnegie Medicine Inc.UK, model CMA/100) was connected to the catheter and air was infused at a rate of 1 ml/min while the colon segment was submerged in PBS. Intraluminal pressure was recorded using a digital manometer (Dwyer Inc. UK, model 477A-4) connected to the silicon catheter using a Y shaped connector. The maximal pressure preceding air leakage from the colon was defined as the colon bursting pressure. The location within the colon in which air leakage occurred was categorized to the anastomosis area vs. extra-anastomotic (outside the sutured area). 

**Assessment of wound healing rate in the skin**

Animals were anesthetized with 2.5% isoflurane, and photographed with a digital camera on Days 2, 4, and 6 following the surgical procedures. The platform upon which animals were positioned, the lightning, the angle of the camera, and the magnification ratio (× 6) were all standardized. The degree of wound healing was scored independently by 4 investigators blind of group assignment, using a 5-rank scale for each of the images in all animals. Results were normalized, and averaged across investigators to generate a wound healing score for each day. 

## Materials 

### Drug selectivity

**COX inhibitors and etodolac**

Warner et al. [67] showed that compared to the selectivity of aspirin (which is 4-fold toward COX-1 vs. COX-2), a group of COX inhibitors, including etodolac, meloxicam, and nimesulide show preferential selectivity toward COX-2 (5 – 50-fold toward COX-2). A second group of COX inhibitors inhibits COX-2 with only a very weak activity against COX-1 (above 50-fold toward COX-2). This group includes rofecoxib, which was withdrawn from clinical use. It should be noted that celecoxib selectivity toward COX-2 inhibition places it in the first group, although its selectivity toward COX-2 is less potent then etodolac. 

**Propranolol**

To block b-adrenoceptor stimulation, we used the non-selective b-adrenergic blocker, propranolol (Sigma, Israel), which was shown effective in our previous studies and in the dose ranges used herein [9]. 

### Drugs and their administration

All animals were randomly assigned into 4 groups based on perioperative drug treatment. Group A received etodolac and vehicle (placebo) of propranolol, Group B received propranolol and vehicle of etodolac, Group C received both active drugs, and Group D had both vehicles only. 

**Etodolac**

The COX-2 inhibitor, etodolac, (Taro, Israel) was dissolved in corn oil. The drug was administered subcutaneously on a daily basis, starting 1 day prior to the surgical procedure, and up to the 7th postoperative day, depending on time of sacrifice. As the half-life time of etodolac in rats was reported to be 18 h [44], a dose of 12 mg/kg was used for the first injection, and a dose of 8 mg/kg for all subsequent injections. These doses are based on prior studies indicating their efficacy in blocking postoperative immunosuppression and tumor progression [9]. 

**Propranolol**

The nonselective b-adrenergic blocker, propranolol (Sigma, Israel) was dissolved in phosphate buffered saline (PBS) and added to a mixture containing mineral oil (Sigma, Israel) and mannide monooleate (Arlacel A, Sigma, Israel), in a 4 : 3 : 1 ratio, respectively, to create a slowly absorbed emulsion. Unpublished data from our laboratory have shown that the slow absorbance emulsion is effective for 36 – 48 h when given in 1 ml volume. The emulsion was administrated subcutaneously 1 day prior to the surgical procedures, on the day of surgery, and each other day postoperatively up to the 7th postoperative day (depending on time of sacrifice), at doses of 0.75 mg/kg, 1.5 mg/kg, and 0.75 mg/kg, respectively (in volumes of 0.5, 1, and 0.5 ml, respectively). The pick dose was found effective in blocking postoperative immunosuppression and tumor progression [9], and the pre and postoperative lower doses were chosen to simulate upscale and weaning of b-blockers in humans. 

### Subjects

**Animals and counterbalancing**

320 (162 male and 158 female) 3 – 4 months old Fischer 344 rats (Harlan laboratories, Jerusalem, Israel), were used. Animals were housed 4 per cage with free access to food and water, and maintained on a 12 : 12 light : dark cycle at 22 °C ± 1. Animals were handled 5 times prior to the experiment in order to reduce procedural stress. The order of drug administration, type of surgical manipulation, and timing of sacrifice were counterbalanced according to the animal’s weight, gender, and the operating surgeon. All studies were approved by The Institutional Animal Care and Use Committee of Tel Aviv University. 

**Statistical analysis**

A 4-way analysis of variance (ANOVA) (2 × 2 × 2 × 3) was used to analyze the dependent variables. The four independent variables were etodolac administration (drug vs. vehicle), propranolol administration (drug vs. vehicle), surgical procedure (anastomosis and punch vs. punch only), and healing interval (e.g., 3, 7, 30 days). The “healing interval” variable was used as a within subject variable in the “skin healing rate” dependent variable, and between subjects in “muscle strength” and “colon bursting pressure” dependent variables. Significant differences between individual groups or specific variables were assessed using Tukey HSD post hoc contrasts. p < 0.05 was considered significant in all assessments. c^2^ was used to compare proportion of animals in which bursting pressure occurred in an extra-anastomotic area, when assessing the impact of different healing intervals or different drug treatments, as well as for testing potential dependency between drug treatments and mortality rates at different time intervals following anastomosis. 

## Results 

A total of 320 rats was included in this study, of which 292 were eligible for study outcomes evaluation per allocation. 28 rats died as a consequence of operation-related complications, as specified below. 

### Early mortality rates and postoperative complications

All 28 rats excluded from the study died within the first 48 postoperative hours. Most of these deaths (24) occurred during or immediately after the operation (within few hours), and resulted from anesthetic complications or major bleeding. The other 4 rats died later within this 48-h period, apparently from surgery-induced bleeding. No signs of anastomotic leak, peritonitis, or intra-abdominal abscess were observed in any case of these 28 deaths. No association between drug treatment and mortality rates was found at this early stage; in fact mortality rate was very similar in all 4 drug/vehicle treatment groups undergoing anastomosis. No mortality occurred beyond the third postoperative day and no leakage was evident in any of the animals when assessing colon bursting pressure at any of the time points studied. 

### Weight loss

Animals undergoing laparotomy and anastomosis showed a 5.7% (SD = 9%) decrease in body weight on Day 5 postoperatively, compared to 1% increase (SD = 1.7%) in the non-operated group. A 3-way ANOVA revealed a main effect for the surgical procedure, but no effects for etodolac, propranolol or their combination on body weight. Variables effecting body weight at later time points were not assessed, as not all groups reached the 5th postoperative day. 

### Abdominal wall tensile strength

**Figure 1:**
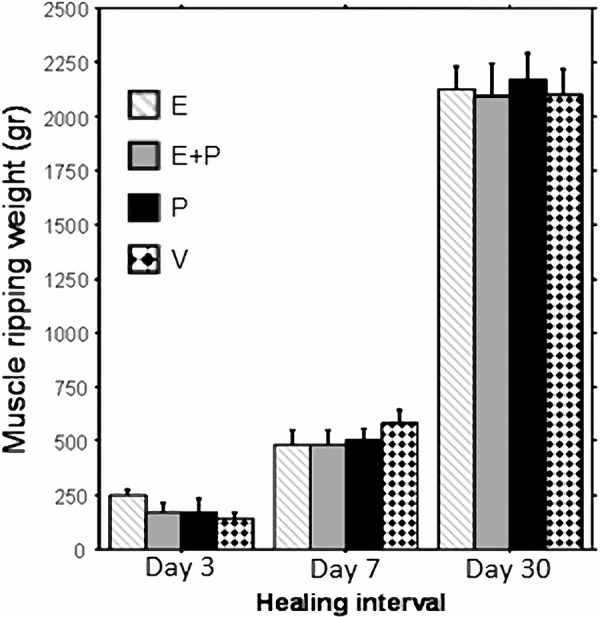
A significant increase in tensile strength postoperatively was observed from Day 3 to 7 (p < 0.0001), and 7 to 30 (p < 0.0001). No effect for any drug treatment was found. Data is presented as Mean ± S.E.M. E = Etodolac, P = Propranolol, V = vehicle

A 3-way ANOVA (2 × 2 × 3) revealed a significant main effect for healing interval (F_(2,134)_ = 465.7, p < 0.0001) (Figure 1). The abdominal wall strength increased significantly from Day 3 to Day 7 (p < 0.0001) and from Day 7 to Day 30 (p < 0.0001). No significant main effect for any of the drug treatments or drugs by healing interval interactions. The surgical procedure variable was not studied, as intact skin and muscles tensile strength in the non-operated groups are not suitable for this assessment. 

### Colon bursting pressure

**Figure 2:**
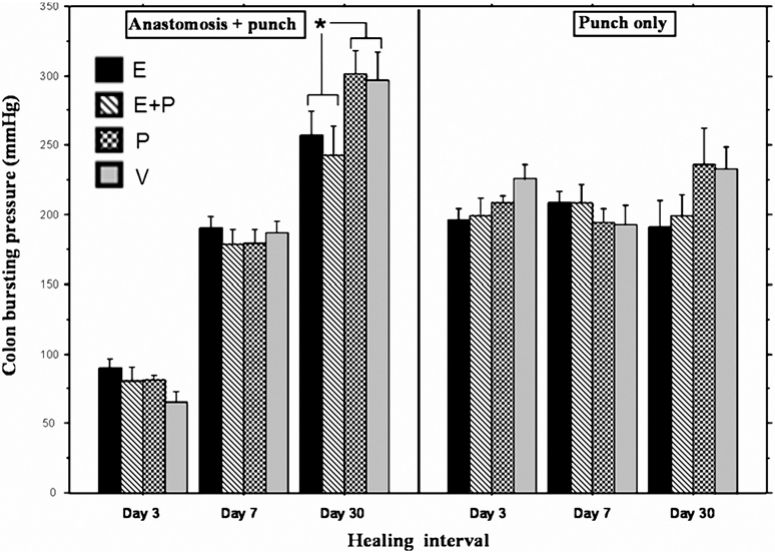
Data is presented as mean bursting pressure ± S.E.M. An anticipated increase in colon bursting pressure was observed from Day 3 to 7, and from Day 7 to 30 postoperatively (p < 0.0001). No significant differences were evident within the non-operated skin punch biopsy groups (punch only). At the 30-day postoperative interval, bursting pressure was significantly higher in animals undergoing anastomosis compared to non-operated animals across drug treatments, and this augmentation was lowered in etodolac treated animals (*p = 0.0036).

On exploration of the abdominal cavity upon sacrifice, no clinical anastomotic leakage was evident in any of the animals. As expected, colon bursting pressure significantly increased from Day 3 to 7 (p < 0.0001) and from Day 7 to 30 (p < 0.0001) in the anastomosis groups. No changes in bursting pressure were evident in groups who did not undergo an anastomosis (punch biopsy only), as indicated by significant interaction between surgical procedure and healing interval (F_(2, 231)_ = 89.69, p < 0.0001) (Figure 2). 

Etodolac, propranolol and their combination did not significantly affect bursting pressure in animals having anastomosis at the 3- and 7-day healing intervals, as well as bursting pressure in non-anastomized colons at all 3 time points. In animals having anastomosis, Tukey HSD showed a significant lower bursting pressure in etodolac-treated groups compared to non etodolac-treated groups at the 30 day interval (p = 0.0036). Nevertheless, this group still had marginally higher bursting pressure when compared to animals who did not have anastomosis at the same time point (p = 0.064). 

Surprisingly, at the 30-day interval, bursting pressure was significantly higher in animals that did have anastomosis compared to non-operated animals. This phenomenon has been observed across all drug treatment groups (Tukey HSD p < 0.0001). 

In animals who had anastomosis, the location of the burst was not in the sutured area in 0% of animals sacrificed 3 days following anastomosis, 20.7% of animals sacrificed 7 days following anastomosis, and 43.8% of animals sacrificed 30 days following anastomosis, yielding a significant main effect for healing interval (c^2^_(2)_ = 27.5, p < 0.001) (not shown). None of the drug treatments impacted this index. 

### Skin healing rate

**Figure 3:**
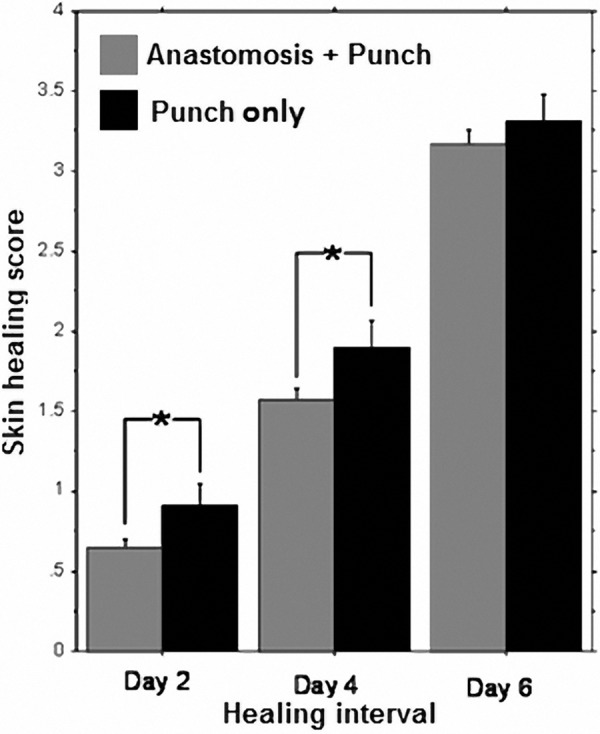
A significant increase in skin healing scores was observed from Day 2 to 4 to 6 postoperatively (p < 0.0001). Operated animals exhibited a significantly lower healing score on Days 2 and 4 postoperatively (*p < 0.0131) compared to non-operated animals, but not on Day 6. No effect for any drug treatment was evident (not shown). Bars show average healing scores per each day and experiment group (punch vs. anastomosis) ± S.E.M.

A 4-way (2 × 2 × 2 × 3) repeated measure ANOVA revealed a significant increase in wound healing rate from Day 2 to 4, to 6 postoperatively (F_(1,158)_ = 506, p < 0.0001) (Figure 3). The variables examined between animals were etodolac administration, propranolol administration and surgical procedure, while healing interval was used as a within animal variable. Approximately half of the animals reached the highest healing score on the 6th day. Thus, to test the effects of the other variables we also ran a separate ANOVA on the 2nd and 4th healing intervals. Laparotomy and anastomosis significantly reduced healing rate (F_(1)_ = 6.23, p < 0.0131), but no drug effects or interactions with drugs were revealed. 

## Discussion 

Surgical and non-surgical stress responses have been shown to suppress various aspects of immunity, specifically cell-mediated immunity (CMI), which plays an important role in anti-metastatic cancer defense [6]. Such suppression was shown to correlate with metastatic recurrence after surgery in patients exhibiting various types of cancer [6]. Several humoral factors have been suggested to promote surgically-induced tumor promotion via immunosuppression, as well as via non-immunological mechanisms. Prominent among these factors are PGs and CAs [6, 66]. 

COX inhibition, through reduction of PG levels, was shown to reduce progression of primary tumors and the development of metastases by several mechanisms, including elevation of postoperative host CMI levels, reduction of tumor-secreted angiogenic agents’ levels [45, 46, 47] and tumor microvascular density [48], and promotion of tumor cell apoptosis [48, 49, 50]. CAs were also implicated in promoting tumor progression. b-adrenoceptors are abundant in leukocytes, and were recently reported to be expressed by several subtypes of human malignancies [51, 52]. CAs were shown to suppress various aspects of CMI [53] and consequently promote tumor metastasis [54]. Additionally, CAs were shown to directly promote tumor cell migration [55, 56, 57], and tumor secretion of pro-angiogenic and pro invasion factors (e.g., Vascular endothelial growth factor (VEGF) and matrix metalloproteinases (MMP)-9/2 [57]), effects that were abolished by b-blockers [51]. 

We have recently shown that the combined treatment of propranolol and etodolac (i) attenuated several postoperative immune perturbations, (ii) increased resistance to metastatic development, and (iii) doubled long-term survival rates in two animal models of postoperative spontaneous metastasis [10] and in a model of leukemia progression [65]. The use of such pharmacologic therapy may be appealing in cancer patients, especially at the perioperative period, where conventional anti-cancer drugs are not usable. 

Before testing such drugs in cancer patients, it is important to assess their impacts on crucial perioperative tissue healing processes. The wound healing process is a highly orchestrated cascade, in which each of the 3 stages, i.e., (i) inflammation, (ii) proliferation, and (iii) remodeling, depends on the successful completion of the preceding stages [12]. When considering patients with cancer of the gastrointestinal tract, an efficient anastomotic wound healing process is crucial, and delayed or deficient healing is highly associated with increased morbidity and mortality [13, 14, 15]. In the current study we tested the effect of treatment with propranolol, etodolac and their combination on wound healing in the colon, muscle, and skin after colonic anastomosis. 

As expected, each of the studied tissues showed a profound time-dependent strengthening. Relatively small effects of the drugs were evident within each time point, as discussed below. Overall, no life-threatening or significant negative effects for any of the drugs’ schedules were observed in any of the tissues examined, and no drug-related mortality occurred in any of the drug schedules. 

As indicated by our results, at the one month healing interval, the operated colon tissue presented strengthening beyond the levels evident in non-operated animals in all drug schedules, and this strengthening seems more pronounced in animals not treated with etodolac. This abnormal strengthening is inconsistent with the common notion that a scar tissue does not surpass 70% of the original tissue strength [58]. However, several previous studies also reported abnormal strengthening associated with surgery, as well as attenuation of this strengthening by COX-2 inhibitors [59, 60], though in different paradigms. It is also noteworthy that in the 3-day healing interval the bursting of the colon never occurred in the extra-anastomotic area of the suture site, while in the week and month healing intervals, 20.7% and 43.8% of the bursting occurred in the extra-anastomotic area (respectively), corresponding with the phenomenon of abnormal strengthening. 

The above impacts of surgery and etodolac on colon tissue strength at the 30-day interval may be explained by two processes. The first process may explain the abnormal strengthening in all surgery groups, and specifically relate to tissue repair in the intestine. During the wound healing period, the intestine resumes functions and needs to cope with pressure generated by peristalsis. Several mechanisms have been suggested to ensure this continued functionality; among them is the formation of a fibrotic ring or a lymphatic plaque around the wound [59], which is believed to strengthen the injured site. This ring or plaque may also account for the 43.8% of animals in which the burst occurred in the extra-anastomotic area at the 30-day healing interval. The second process, which may account for the reduced strengthening in the etodolac administered groups (E & E+P) at the 30-day interval, may be related to a time-dependent impact of PGs on tissue healing. Specifically, blockade of PGE-2 was shown to reduce the inflammatory stage [61], in which neutrophils infiltration is predominant. These neutrophils produce matrix-degrading enzymes, such as MMP, which consume the wounded tissue and its’ surrounding in preparation for regenerative processes. The use of etodolac may reduce degradation of damaged and non-damaged tissue, and reduced area of future scar tissue. Thus, it is possible that reduction in the removal of the wounded area by MMPs during the early inflammatory stage may result in the formation of a smaller and less stable basis for future regeneration. This may account for the smaller abnormal strengthening evident in the etodolac-treated groups at the one month interval period. 

With respect to wound healing of the skin biopsy (punch), no drug effects were evident in operated or non-operated animals. Operated animals showed a small but significant slowing of skin healing. Studies have shown that distal bacterial infection contributes to an inhibitory effect on the skin wound healing process [62]. It has also been showed that stress promotes trafficking of immunocytes between body regions [63], and that psychological stress (e.g., restraint) produces an inhibitory effect on skin wound healing [64]. We propose that the effects of surgery may be attributed to redistribution of immunological resources between multiple wounds, which may lead to reduced skin inflammatory stage and slower healing. This hypothesis is supported by unpublished data from our lab indicating that during stress and following surgery there is a marked reduction in the number of subcutaneous leukocytes [abstract submitted to PNIRS 2010]. This reduction could account for a weaker immune response and a slower healing rate. 

## Conclusions 

The results of this study demonstrate that the use of etodolac and propranolol, combined or separately, do not have major impact on wound healing in parameters pertinent to colon and rectal surgery. Etodolac may have a minor effect on tissue healing, in a time-dependant manner, with questionable clinical relevance. Importantly, none of the drug schedules seemed to exhibit any clinically-relevant deleterious effect, suggesting the safety of their potential use in colorectal cancer patients. 

## Acknowledgments 

This work was supported by NIH/NCI grant #CA125456 (SBE), and a grant from the Israel-USA Bi-National Science Foundation #2005331 (SBE).
